# Age-Period-Cohort Analysis of HIV Mortality in China: Data from the Global Burden of Disease Study 2016

**DOI:** 10.1038/s41598-020-63141-1

**Published:** 2020-04-27

**Authors:** Disi Gao, Zhiyong Zou, Wenjing Zhang, Tianqi Chen, Wenxin Cui, Yinghua Ma

**Affiliations:** Institute of Child and Adolescent Health, School of Public Health, Peking University Health Science Center, Beijing, China

**Keywords:** HIV infections, Epidemiology

## Abstract

The aim of this study was to investigate the long-term trends of human immunodeficiency virus (HIV) mortality in China and its associations with age, period and birth cohort. We used HIV mortality data obtained from the Global Burden of Disease Study (GBD) 2016 and analysed the data with an age-period-cohort framework. Age effects indicate different risks of different outcomes at specific periods in life; period effects reflect population- wide exposure at a circumscribed point in time; and cohort effects generally reflect differences in risk across birth cohorts.Our results showed that the overall annual percentage change (net drift) of HIV mortality was 11.3% (95% CI: 11.0% to 11.6%) for males and 7.2% (95% CI: 7.0% to 7.5%) for females, and the annual percentage changes in each age group (local drift) were greater than 5% (p < 0.01 for all) in both sexes. In the same birth cohort, the risk of death from HIV increased with age in both sexes after controlling for period effects, and the risk for each five-year period was 1.98 for males and 1.57 for females compared to their previous life stage. Compared to the period of 2002–2006, the relative risk (RR) of HIV mortality in 2012–2016 increased by 56.1% in males and 3.7% in females, and compared to the 1955–1959 birth cohort, the cohort RRs increased markedly, by 82.9 times in males and 34.8 times in females. Considering the rapidly increasing risk of HIV mortality, Chinese policymakers should take immediate measures to target the key age group of 15–44 years in both sexes.

## Introduction

Human immunodeficiency virus (HIV) and the consequent acquired immune deficiency syndrome (AIDS) have caused one of the worst epidemics affecting humanity since the late 20th century, leading to a substantial number of deaths^1,2^. In 2018, 770,000 people died from AIDS-related diseases; the Eastern and southern Africa regions had the highest numbers of AIDS-related deaths, accounting for a combined 40.3%, followed by Asia and the Pacific region, accounting for 25.0%^3^. From 2010 to 2018, the number of global AIDS-related deaths decreased by 33%; however, the HIV mortality ratio increased from 1.54 per 100,000 to 1.71 per 100,000 individuals^3^. HIV/AIDS has also become the leading infectious cause of death in many countries^4^. In South Africa, from 1992 to 2013, more than 60% of deaths were attributed to HIV^5^. In Russia, HIV/AIDS has been the fastest increasing cause of premature death in the past decade, with an 86.5% increase^6^.

HIV became the leading infectious cause of death in China in 2009, and the number of deaths due to HIV/AIDS increased from 5,544 in 2007 to 21,234 in 2011^7,8^. In addition, HIV mortality in males is much higher than that in females in China^9^. An existing study showed that the HIV prevalence in China increased exponentially over the past 16 years, and HIV mortality increased from 0.0002 per 100,000 individuals in 1992 to 0.9362 per 100,000 individuals in 2016^10^. However, previous studies mainly focused on the age distribution of the incidence or mortality, and period and cohort effects were not taken into account. The trends of HIV mortality in different age groups remain unclear, and the relative risk (RR) due to period and cohort effects remains unknown. Therefore, a comprehensive analysis to address these limitations and elucidate answers to these questions was necessary.

In this study, we used data from the Global Burden of Disease Study (GBD) 2016 to investigate the long-term trends in HIV mortality in China and to examine the contributions of age, period, and cohort effects from 1992 and 2016. The findings from our study may provide guidance for resource allocation to prevent HIV-related deaths in vulnerable target populations.

## Data sources and methods

### Data sources

The data used in this study were extracted from the GBD 2016, a large international cooperative project that globally, regionally, and nationally assessed age-sex mortality for 264 causes of death, including HIV, from 1980 to 2016^11^. Original data, which the GBD adapted to estimate HIV mortality, were mainly obtained from the Disease Surveillance Point (DSP) and the Notifiable Infectious Disease Reporting (NIDR) systems. Both systems are administered by the Chinese Center for Disease Control^11^. We also used data from the GBD 2013 global age-standardized population to standardize the HIV mortality rate for both males and females^12^.

### Statistical analyses

Age-period-cohort (APC) analysis was used to evaluate the effects of age, period and cohort on the disease rate outcomes. Age effects indicate the different risks of different outcomes during different periods of life; period effects reflect population-wide exposure at a circumscribed point in time; and cohort effects generally represent the differences in risk across birth cohorts^13,14^.By decomposing the age and cycling a queue into their linear and nonlinear parts^15^, we can not only avoid directly separating the contributions of age, period and cohort^15^ but also estimate many useful functions, such as net drift; longitudinal age trend; and age, period and cohort deviations^14,16^. The net drift indicates the overall annual percentage change adjusted for age group over time, and local drifts indicate annual percentage changes for each age group. The longitudinal age curve, which is adjusted for period deviations, indicates the fitted longitudinal age-specific rates in the reference cohort. The period RR indicates the period RR adjusted for age and nonlinear cohort effects in each period relative to the reference period, and the cohort RR indicates the cohort RR adjusted for age and nonlinear period effects in each cohort relative to the reference period. Although the APC model has advantages in analysis, it also has unique and unfortunate limitations, including identifiability problems and uncertainty principles. The identifiability problem refers to the fact that the three time scales of age, period and cohort are collinear (cohort equals period minus age); therefore, the log-linear trends in rates cannot uniquely be attributed to the influences of age, period, or cohort^17^. The uncertainty principle refers to the measurement of absolute rates in cohorts, which is rarely considered in the context of most epidemiological cohort and case-control studies^18^.

To conduct the APC analysis, the mortality and population data were arranged into consecutive 5-year periods from 1992–1996 (median 1994) to 2012–2016 (median 2014) (data from 1990 to 1992 were not considered because they were not sufficient for a 5-year period); successive 5-year age groups from 15–19 years to 75–79 years (individuals younger than 15 years and older than 80 years were excluded); and 17 consecutive cohorts, including those born from 1915–1919 (median 1917) to 1990–1994 (median 1992). We used the APC Web Tool (Biostatistics Branch, National Cancer Institute, Bethesda, MD, USA) to obtain these estimable parameters. In all APC analyses, the central age group, period, and birth cohort were defined as the reference. The reference value was set as the lower of the 2 central values in the event of an even number of categories. We used Wald chi-square tests to calculate the significance of the estimable parameters and functions. A general linear model was used to compare the significance of the slope of the period RR and cohort RR by checking the interaction effect between sex and calendar year/birth cohort^19^. All statistical tests were 2-sided, and p-values less than 0.05 were considered statistically significant.

## Results

**Figure 1:**
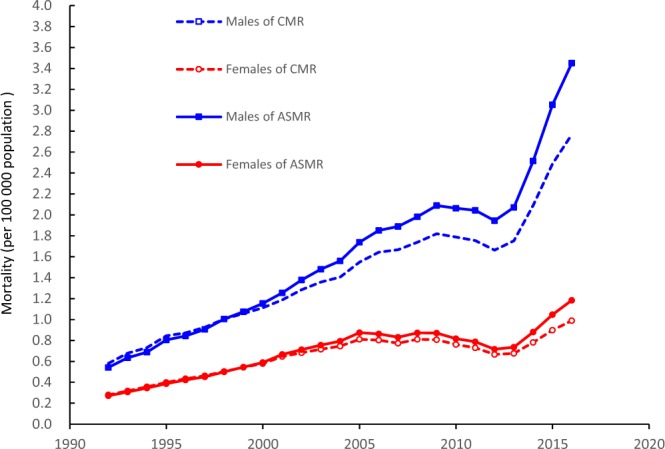
Trends in the HIV CMRs and the ASMRs per 100,000 population by sex from 1992 to 2016 using the GBD 2013 global age-standardized population.

Figure 1 shows the trends in crude mortality rates (CMRs) and the age-standardized mortality rates (ASMR) for HIV by sex for the period of 1992 to 2016. The HIV CMRs showed generally increasing trends in both sexes, from 0.58 to 2.77 per 100,000 individuals for males and 0.28 to 0.99 per 100,000 individuals for females, and there was a slight decrease from 2010 to 2013. The ASMRs in both sexes were similar to the trends in the CMRs, the ASMR increased 6.4-fold for males and 4.4-fold for females over the past 25 years. Our results also indicated that the ASMR of HIV of in males increased much faster than that in females, and in 2016, the male ASMR was nearly three times that of females.

**Figure 2:**
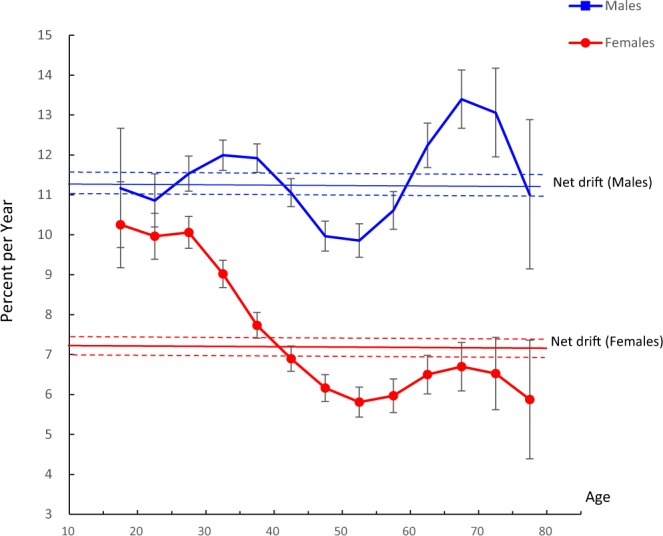
Local drift and net drift values for HIV mortality in China. Age group-specific annual percentage change (local drift) with the overall annual percentage change (net drift) in the HIV mortality rate and the corresponding 95% confidence intervals (some of them were too narrow to show in the figure).

The net drift and local drifts are displayed in Figure 2. From 1990 to 2016, the net drifts were 11.3% (95% CI, 11.0% to 11.6%) per year for males and 7.2% (95% CI, 7.0% to 7.5%) per year for females, indicating that the overall annual percentage increase in HIV mortality in males was nearly 1.6 times that in females. The local drift values were above 0 for all age groups in both sexes and in each age group. Rapidly increasing trends in HIV mortality were observed in males aged 15–44 years and 65–74 years and in females aged 15–44 years. The mortality rate increased from 10.9% to 12.0% in the 15–44 years age group and from 12.2% to 13.4% in the 65–74 years age group in males and from 6.9% to 10.3% in the 15–44 years age group females aged.

Table S1 shows the trends in HIV/AIDS mortality in China from 1992 to 2016, and Table S2 shows the numbers of HIV/AIDS deaths across each age period. From 1992 to 2016, the HIV/AIDS mortality rate in males experienced a significant growth, especially in the 15–44 years age group and 65–74 years age group. For example, males aged 65–69 (median 67) had HIV/AIDS mortality rates of 0.38, 0.55, 1.12, 1.22 and 1.25 per 100,000 individuals during the periods of 1994, 1999, 2004, 2009 and 2014, respectively. The HIV/AIDS mortality rate in females also increased between 1992 and 2016, and the growth of the HIV/AIDS mortality rate for females was highest in the 15–19 year age group (median 17), from 0.02 per 100,000 individuals in 1992–1996 (median 1994) to 0.15 per 100,000 individuals in 2012–2016 (median 2014).

**Figure 3:**
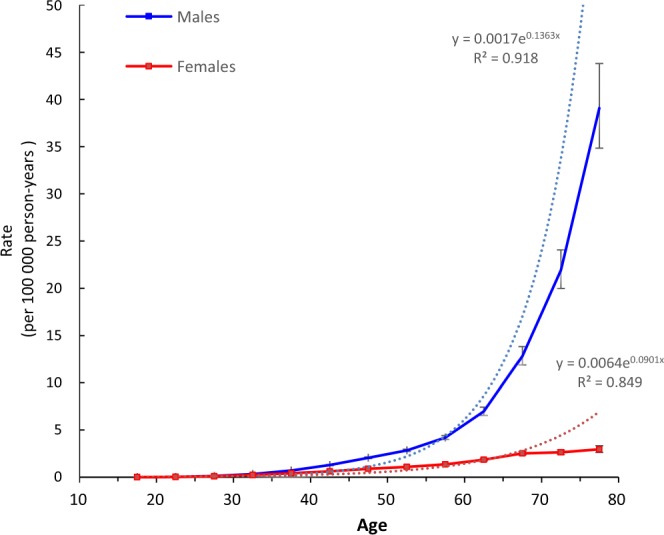
Longitudinal age curves of HIV mortality in China. Fitted longitudinal age-specific rates of HIV mortality (per 100 000 person-years) and the corresponding 95% confidence intervals (some of them were too narrow to show in the figure).

Figure 3 illustrates the longitudinal age curve of HIV mortality by sex adjusted for period. In the same birth cohort, the mortality risk in both males and females increased with age, and for males, the mortality risk showed an accelerated increase. There was a striking difference between males and females, and the risk of death from HIV in males was much higher than that in females, especially for people older than 40 years. The mortality risk for males in this age group was 6–32 times that for females in this group. We also estimated the longitudinal age curves and found that the male curve followed an exponential distribution and the female curve followed a linear distribution. The curves can be expressed as rate=0.0017*e^0.1363* mean_age^ for men (R-square=0.918) and rate=0.0064*e^0.0901* mean_age^ for females (R-square=0.849), indicating that the RR for HIV mortality in each life stage from the 20–24 years of age to 75–79 years of age was 1.98 for males and 1.57 for females compared to their previous life stage.

**Figure 4:**
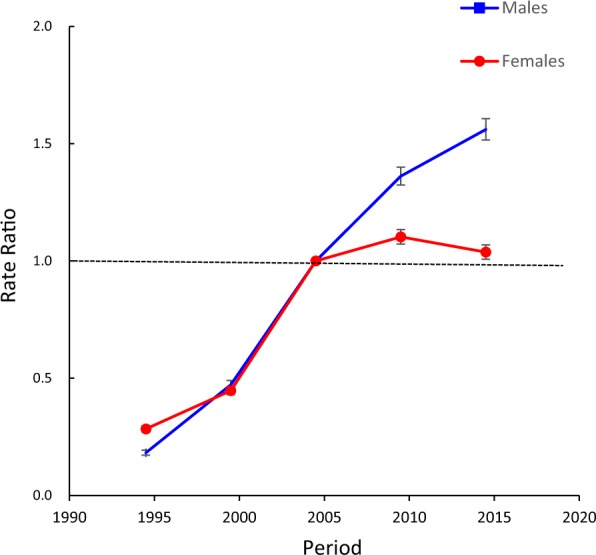
Period RRs of HIV mortality by sex in China. Period effects obtained from APC analyses for HIV mortality rates and the corresponding 95% confidence intervals (some of them were too narrow to show in the figure) by sex in China.

**Figure 5:**
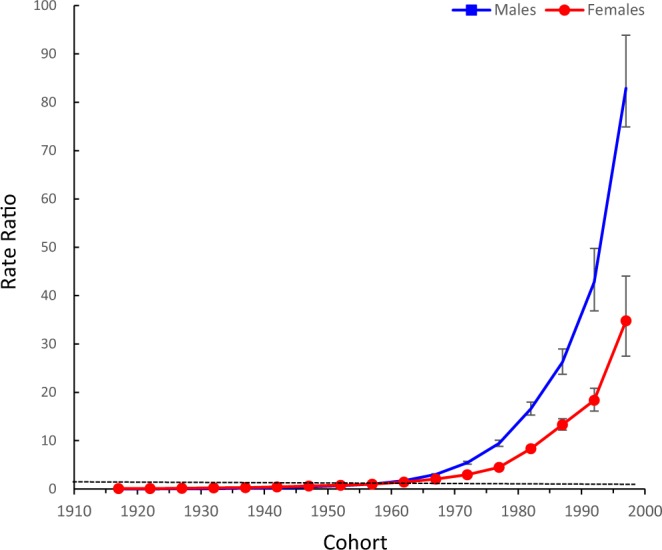
Cohort relative risks (RRs) of HIV mortality rates by sex in China. Cohort effects obtained from APC analyses for HIV mortality rates and the corresponding 95% confidence intervals (some of them were too narrow to show in the figure) by sex in China.

Figures 4 and 5 show the estimated period and cohort RRs by sex. The period RRs were found to have similar monotonically increased patterns in both sexes, with more rapid increases in males than in females after 2005. The cohort RRs showed similar monotonic increasing patterns in both sexes, and the increasing trend in males was accelerated in the cohort born in the 1980s. Compared to the period of 2002–2006, the period RRs of HIV mortality in 2012–2016 increased by 56.1% in males and 3.7% in females, and compared to the 1955–1959 birth cohort, the cohort RRs markedly increased by 82.9 times for males and by 34.8-fold in females. In addition, based on the specific results of the Wald tests, there were statistically significant cohort and period RRs for both sexes (p < 0.01 for all), and the net drift and local drifts were also statistically significant (p < 0.01 for all).

## Discussion

Our study explored the long-term trends in HIV mortality in China between 1992 and 2016 with the aid of an APC framework; to our knowledge, this is the first study to investigate APC-specific effects of HIV mortality by sex. Our results indicated that HIV mortality in China increased rapidly, especially in males. In addition, the percentage change in males aged 15–44 years and aged 65–74 years and in females aged 15–44 years also increased dramatically during the period from 1992 to 2016.

In theory, with the development of antiretroviral treatment (ART), the HIV mortality rate should have decreased; however, the present study showed that HIV mortality in China continued to rise from 1992 to 2016. In China, the low perception of risk^20^ and low rates of HIV testing (especially among key populations, such as men who have sex with men, MSM)^21^ are likely to have contributed to this increasing trend, which was similar to the general trend for all of Asia^22^. The net drift of HIV mortality in both sexes also indicated that the risk of HIV mortality increased in all age groups, and the reasons why HIV mortality among males was higher than that among females may be as follows. First, MSM are at a much higher risk of HIV infection than females^23–26^. Second, females benefit more than males from HIV treatment, which helps to reduce the HIV death rate^27–29^. Third, the sex distribution in China is unbalanced; from 1990 to 2014, the sex ratio (male vs female ratio) at birth (SRB) was greater than 110^30^.

The local drift results also indicated that HIV mortality in those aged 15–44 years in both sexes and in males aged 65–74 years showed a significant increase over the past 25 years; these groups have been identified as target groups for HIV prevention and treatment. These results were similar to those reported in some provinces in China^31–33^. The reasons for the increase in HIV/AIDS mortality among females aged 15–44 years may be as follows. First, with the continuous spread of HIV in China^10^, the mortality rate of HIV/AIDS has also increased rapidly, including in females aged 15–44 years. Second, the average age of first intercourse in Chinese adolescents is 18–20 years^34^, and considering the characteristics of adolescents and their frequency of sexual behaviour, they usually pursue high-intensity stimulation without condom use, which increases the risk of HIV infection^35^. The survival period (from diagnosis to death) of HIV patients in China is 5 to 20 years;^36–39^ therefore, when young females are infected with HIV at a young age, they will likely die within the following 20 years. Third, late HIV diagnosis is a severe public health problem in China^40,41^, suggesting that as epidemic HIV/AIDS expands in China, an increasing number of HIV-infected people are not diagnosed early. Chinese studies have found that more than 1/3 of HIV-infected patients had CD4 counts of<200 cells/µL at diagnosis;^41–43^ another Chinese study found that more than 2/3 of patients died within one year after diagnosis^44^. HIV infection via heterosexual behaviour has a high rate of late diagnosis, and most females are infected during heterosexual encounters; thus, their rate of late HIV diagnosis is high^45^. Therefore, the increase in HIV mortality in females aged 15–44 years in this study is likely due to the increase in late HIV diagnosis, females diagnosed at later stages of infection missed the optimal time for ART and often due of HIV/AIDS-related diseases at a very young age.

Age is one of the most important demographic factors that affects HIV mortality, and many surveys^40,46^ have shown that age over 40 years is strongly associated with AIDS-related disease mortality. Our research also showed that in the same birth cohort, the risk of HIV mortality increased rapidly with age after adjusting for period deviations, especially in males; however, for females, the RR of HIV mortality in the older age group remained relatively low. The reasons for these findings may be as follows. First, the number of infected females was much lower than the number of infected males^47^. Second, among individuals who receive HIV treatment, females have a longer life expectancy than males^28^.

In this study, we separately estimated the period effect and cohort effect in the APC model; however, interpreting these models separately in the real world is difficult. On the one hand, the period effect may influence certain age groups differently and can lead to cohort effects. On the other hand, individuals from different cohorts were born in different periods, which inevitably has a confounding impact on period effects to some extent. Therefore, we systematically analysed the possible reasons for the increasing trends in the period and cohort effects. The most probable reasons that explain the increase in HIV mortality and HIV mortality RRs in China over the last 25 years are as follows. First, when HIV was identified in China in 1985^48^, China was in a period of sexual liberation^49^. Chinese people gradually abandoned the traditional concept of sex and recognized premarital sex. However, at that time, sex education was not popularized in China (even now it still imperfect), and the majority of HIV transmission was through sexual intercourse; therefore, many Chinese people were infected with HIV during this time. Second, although the introduction of ART has helped many HIV-infected people in China^50^, highly active antiretroviral therapy (HAART), late diagnosis, poverty, low education status and other reasons have been demonstrated to be strongly related to AIDS-associated deaths^46^. Therefore, to prevent the risk of death caused by HIV/AIDS, HIV prevention should be a priority.

HIV preexposure prophylaxis (PrEP) has been proven to be an effective tool to reduce HIV transmission, especially transmission via sexual practices among MSM^51–53^, but it is often underutilized^54,55^. The reasons for the underutilization of PrEP include lack of knowledge of the drug or concern about its side effects^56,57^. Medical providers play an important role in advising high-risk HIV/AIDS groups about PrEP, and this education should lead to improved utilization rates^51^.Therefore, it is necessary to emphasize the role of medical staff in patient education. Post-exposure prophylaxis (PEP) is another successful method to prevent HIV infection after exposure^58^, but it’s utilization also faces many challenges, such as failure to access the agent in time or false-negative results^59,60^. It has been proven that simple collaborative interventions and improved communication and coordination among different departments (including the pharmacy department and infectious disease department) can effectively promote PEP to reduce critical delays in HIV prevention in vulnerable populations^59^. As PrEP and PEP play a significant role in the prevention of HIV transmission, the Chinese government should consider both methods together with ART when developing strategies to prevent the spread of HIV and reduce HIV/AIDS-related deaths.

Our study has some limitations. First, this study did not include individuals under 15 years and over 80 years old in the analysis because HIV mortality in those under 15 years old is very rare, and individuals over 80 years old were recorded as only one group (an all-ages group) in the GBD 2016 database. Although these age groups made up only a small proportion and did not influence our conclusion, we still cannot neglect them, and according to our prediction, the HIV mortality rate among elderly individuals over 80 years old is still increasing. Second, our study has an ecological fallacy and unique limitations associated with the APC model (including the identifiability problem and uncertainty principle); these limitations were inevitable because the interpretations of results at the population levels do not necessarily hold true at the individual level. Many other studies focused on the trends of cardiovascular disease mortality or cancer mortality using the same web tool for the APC model, similar to our study^13,61,62^. Therefore, future large-scale cohort studies are needed to confirm the related hypotheses in this study.

## Conclusions

The ASMR of HIV in China increased dramatically from 1992 to 2016, especially in males. Considering the rapidly increasing risk of HIV mortality in young people, Chinese policymakers should take immediate measures, such as HIV promotion and education about PrEP and PEP, increasing HIV testing rate and surveillance and urging infected persons to receive ART as soon as possible to prevent HIV infection and reduce the mortality rate, to target the key age group of 15 to 44 years.

## Supplementary information

S1 Table.

S2 Table.

## Data Availability

The data set supporting the conclusions of this article are available in the GBD Data Tool repository (http://ghdx.healthdata.org/gbd-2016).
