# Supplementary material for: Age-Period-Cohort Analysis of HIV Mortality in China: Data from the Global Burden of Disease Study 2016
Source: Sci Rep. 2020 Apr 27;10:7065. doi: 10.1038/s41598-020-63141-1 (PMC7184615; doi:10.1038/s41598-020-63141-1)
Supplement: Supplementary file 1 — S1 Table. [file 41598_2020_63141_MOESM1_ESM.pdf]

Age-Period-Cohort Analysis of HIV Mortality in China: Data from the Global Burden of Disease Study 2016

Disi Gao[†1], Zhiyong Zou[†1], Wenjing Zhang[1], Tianqi Chen[1], Wenxin Cui[1]， Yinghua Ma[1*]

S1 Table. Crude mortality rate of HIV/AIDS by age, period and median birth cohorts in China, 1992 to 2016 (per 100,000 population)

**Median Period**	**Age group**													**Median Birth Cohort**
	**15-19**	**20-24**	**25-29**	**30-34**	**35-39**	**40-44**	**45-49**	**50-54**	**55-59**	**60-64**	**65-69**	**70-74**	**75-79**	
Male													0.507	1917
												0.369	0.970	1922
											0.413	0.907	1.846	1927
										0.444	0.997	2.090	2.955	1932
									0.492	1.097	2.236	3.155	4.082	1937
								0.561	1.247	2.390	3.364	4.227		1942
							0.627	1.412	2.628	3.319	4.522			1947
						0.603	1.557	2.757	3.214	3.633				1952
					0.507	1.424	2.966	3.234	3.073					1957
				0.436	1.389	3.166	4.008	3.632						1962
			0.261	1.149	2.938	4.314	4.612							1967
		0.087	0.738	2.518	4.286	5.216								1972
	0.011	0.247	1.618	3.519	4.966									1977
1994	0.031	0.538	2.258	4.146										1982
1999	0.099	0.729	2.324											1987
2004	0.142	0.744												1992
2009	0.165													
2014														

Median	Age group													Median
**Period**	**15-19**	**20-24**	**25-29**	**30-34**	**35-39**	**40-44**	**45-49**	**50-54**	**55-59**	**60-64**	**65-69**	**70-74**	**75-79**	**Birth Cohort**
Female													0.254	1917
												0.265	0.356	1922
											0.377	0.410	0.711	1927
										0.390	0.551	0.845	0.766	1932
									0.375	0.571	1.116	0.918	0.724	1937
								0.407	0.577	1.189	1.222	0.951		1942
							0.456	0.632	1.225	1.231	1.246			1947
						0.361	0.659	1.283	1.254	1.125				1952
					0.347	0.546	1.316	1.297	0.991					1957
				0.299	0.554	1.326	1.429	1.153						1962
			0.185	0.454	1.238	1.474	1.337							1967
		0.072	0.289	1.009	1.382	1.404								1972
	0.017	0.121	0.719	1.196	1.410									1977
1994	0.032	0.363	1.002	1.518										1982
1999	0.106	0.458	1.061											1987
2004	0.130	0.392												1992
2009	0.146													
2014														

The cohort follows from lower left to upper right, going diagonally. See S2 table for numbers of HIV/AIDS death for each of age-period cross classification. All data were

obtained from the GBD Results Tools (http://ghdx.healthdata.org/gbd-2016)
