# Supplementary material for: Age-Period-Cohort Analysis of HIV Mortality in China: Data from the Global Burden of Disease Study 2016
Source: Sci Rep. 2020 Apr 27;10:7065. doi: 10.1038/s41598-020-63141-1 (PMC7184615; doi:10.1038/s41598-020-63141-1)
Supplement: Supplementary file 2 — S2 Table. [file 41598_2020_63141_MOESM2_ESM.pdf]

# Age-Period-Cohort Analysis of HIV Mortality in China: Data from the Global Burden of Disease Study 2016

Disi Gao[†1], Zhiyong Zou[†1], Wenjing Zhang[1], Tianqi Chen[1], Wenxin Cui[1], Yinghua Ma[1*]

S2 Table. Numbers of HIV/AIDS deaths by age, period and median birth cohorts in China, 1992 to 2016

| Median Period | Age group | | | | | | | | | | | | | Median Birth Cohort |
|---|---|---|---|---|---|---|---|---|---|---|---|---|---|---|
| | 15-19 | 20-24 | 25-29 | 30-34 | 35-39 | 40-44 | 45-49 | 50-54 | 55-59 | 60-64 | 65-69 | 70-74 | 75-79 | |
| Male | | | | | | | | | | | | | 756 | 1917 |
| | | | | | | | | | | | | 874 | 1580 | 1922 |
| | | | | | | | | | | | 1455 | 2538 | 3654 | 1927 |
| | | | | | | | | | | 2136 | 4199 | 7172 | 7347 | 1932 |
| | | | | | | | | | 2804 | 5792 | 10500 | 12272 | 11681 | 1937 |
| | | | | | | | | 3468 | 7368 | 13198 | 16702 | 17487 | | 1942 |
| | | | | | | | 4765 | 10455 | 18713 | 22280 | 27375 | | | 1947 |
| | | | | | | 6393 | 16243 | 28098 | 31689 | 33865 | | | | 1952 |
| | | | | | 5609 | 15582 | 31922 | 34096 | 31374 | | | | | 1957 |
| | | | | 5602 | 17680 | 39790 | 49681 | 44181 | | | | | | 1962 |
| | | | 4142 | 18099 | 45784 | 66533 | 70265 | | | | | | | 1967 |
| | | 1405 | 11818 | 39947 | 67405 | 81306 | | | | | | | | 1972 |
| | 152 | 3272 | 21230 | 45787 | 64137 | | | | | | | | | 1977 |
| 1994 | 398 | 6848 | 28518 | 51997 | | | | | | | | | | 1982 |
| 1999 | 1611 | 11790 | 37368 | | | | | | | | | | | 1987 |
| 2004 | 2199 | 11059 | | | | | | | | | | | | 1992 |
| 2009 | 1845 | | | | | | | | | | | | | |
| 2014 | | | | | | | | | | | | | | |

| **Median Period** | **Age group** | | | | | | | | | | | | | **Median Birth Cohort** |
|---|---|---|---|---|---|---|---|---|---|---|---|---|---|---|
| | **15-19** | **20-24** | **25-29** | **30-34** | **35-39** | **40-44** | **45-49** | **50-54** | **55-59** | **60-64** | **65-69** | **70-74** | **75-79** | |
| Female | | | | | | | | | | | | | 469 | 1917 |
| | | | | | | | | | | | | 713 | 700 | 1922 |
| | | | | | | | | | | | 1370 | 1181 | 1679 | 1927 |
| | | | | | | | | | | 1776 | 2170 | 3013 | 2124 | 1932 |
| | | | | | | | | | 1936 | 2736 | 4986 | 3558 | 2261 | 1937 |
| | | | | | | | | 2420 | 3092 | 6218 | 5808 | 3972 | | 1942 |
| | | | | | | | 3635 | 4202 | 8672 | 7939 | 7578 | | | 1947 |
| | | | | | | 3588 | 6055 | 12567 | 11677 | 10338 | | | | 1952 |
| | | | | | 3823 | 5781 | 13690 | 13131 | 9809 | | | | | 1957 |
| | | | | 3875 | 6427 | 16264 | 16809 | 13502 | | | | | | 1962 |
| | | | 2799 | 6524 | 18652 | 21612 | 19782 | | | | | | | 1967 |
| | | 1046 | 4448 | 15023 | 20777 | 21002 | | | | | | | | 1972 |
| | 207 | 1611 | 8879 | 15083 | 17322 | | | | | | | | | 1977 |
| 1994 | 394 | 4476 | 12067 | 18066 | | | | | | | | | | 1982 |
| 1999 | 1581 | 6752 | 15869 | | | | | | | | | | | 1987 |
| 2004 | 1768 | 5277 | | | | | | | | | | | | 1992 |
| 2009 | 1444 | | | | | | | | | | | | | |
| 2014 | | | | | | | | | | | | | | |

The cohort follows from lower left to upper right, going diagonally.
